# Associations of production characteristics with the on-farm presence of *Fasciola hepatica* in dairy cows vary across production levels and indicate differences between breeds

**DOI:** 10.1371/journal.pone.0294601

**Published:** 2023-11-17

**Authors:** Andreas W. Oehm, Yury Zablotski, Martina Hoedemaker, Amely Campe, Christina Strube, Daniela Jordan, Andrea Springer, Markus Klawitter, Gabriela Knubben-Schweizer

**Affiliations:** 1 Institute of Parasitology, Vetsuisse Faculty of Zurich, University of Zurich, Zurich, Switzerland; 2 Clinic for Ruminants with Ambulatory and Herd Health Services, Ludwig-Maximilians-Universität Munich, Oberschleissheim, Germany; 3 Clinic for Cattle, University of Veterinary Medicine Hannover Foundation, Hannover, Germany; 4 Department of Biometry, Epidemiology and Information Processing, University of Veterinary Medicine Hannover, Hannover, Germany; 5 Institute for Parasitology, Centre for Infection Medicine, University of Veterinary Medicine Hannover, Hannover, Germany; 6 Clinic for Ruminants and Swine, Faculty of Veterinary Medicine, Freie Universität Berlin, Berlin, Germany; Visayas State University, PHILIPPINES

## Abstract

*Fasciola hepatica* is one of the economically most important endoparasites in cattle production. The aim of the present work was to evaluate the relevance of production level on the associations of on-farm presence of *F*. *hepatica* with farm-level milk yield, milk fat, and milk protein in Holstein cows, a specialised dairy breed, and in Simmental cows, a dual purpose breed. Furthermore, we investigated whether differential associations were present depending on breed. Data from 560 dairy farms across Germany housing 93,672 cows were analysed. The presence of *F*. *hepatica* antibodies was determined via ELISA on bulk tank milk samples. Quantile regression was applied to model the median difference in milk yield, milk fat, and milk protein depending on the interaction of breed and fluke occurrence. Whereas a reduction in milk yield (-1,206 kg, p < 0.001), milk fat (-22.9 kg, p = 0.001), and milk protein (-41.6 kg, p <0.001) was evident on *F*. *hepatica* positive German Holstein farms, only milk fat (-33.8 kg, p = 0.01) and milk protein (-22.6 kg, p = 0.03) were affected on *F*. *hepatica* positive German Simmental farms. Subsequently, production traits were modelled within each of the two breeds for low, medium, and high producing farms in the presence of *F*. *hepatica* antibodies and of confounders. On Holstein farms, the presence of *F*. *hepatica* seropositivity was associated with lower production, while on German Simmental farms such an association was less evident. This work demonstrates that production level is relevant when assessing the associations between the exposure to *F*. *hepatica* with production characteristics. Moreover, both models indicate a breed dependence. This could point towards a differential *F*. *hepatica* resilience of specialised dairy breeds in comparison with dual purpose breeds.

## Introduction

In light of the increased interest in animal welfare and sustainability of the food supply chain, sustainable livestock managing practises have become paramount to advance preservation of natural resources and to promote the wellbeing of farmed animals [1]. Maintaining and improving the health of livestock is crucial for minimising emission intensity of farming, for optimising productivity, and for ensuring animal welfare. The efficiency and sustainability of livestock production yet is substantially compromised by endemic diseases that constrain the health, welfare, and viability of farmed animals [2–4]. The common liver fluke, *Fasciola hepatica*, has been regarded as one of the economically most relevant parasitic helminths in ruminant production with an estimated global economic impact of several billion USD per year [5, 6]. Production losses due to bovine fasciolosis are attributable to decreases in milk yield and milk quality [7, 8], reduced weight gain [7, 9], impaired reproductive performance [7, 10], costs for preventive and therapeutic interventions [7, 10] as well as to secondary bacterial infections in the course of immunomodulatory effects of the parasite [11, 12]. Specifically, Schweizer et al. [7] estimated a mean reduction of 9% in weight gain in growing cattle, a decrease in milk yield of 10% as well as a service period extended by 13 days, and an increase of 0.75 services per conception. According to recent work, the economic losses due to fasciolosis are likely to become even more pronounced when acknowledging climate change-induced effects and a higher proportion of herds possibly becoming exposed to *F*. *hepatica* [13]. Interestingly, notwithstanding the similar impact of fasciolosis across farms, some operations appear to be more capable of adjusting their systems and thus mitigating the impact of the disease, whereas others are more vulnerable to the adverse implications of fasciolosis [13].

In sheep, resilience to helminth infection, defined as the capability of animals to thrive in the face of infection [14], has been documented as early as 1987 [15]. In cattle however, equivocal information has been presented. Whereas Twomey et al. [16] were able to acknowledge only little or no variability in resilience among cattle to *Fasciola*, anecdotal evidence has suggested reduced parasite burdens or lower levels of egg excretion in certain indigenous ruminant breeds [4, 17, 18]. This is supported by recent findings of higher fluke burdens in animals of the Friesian breed compared with Jersey cows [19] and of a negative association between *F*. *hepatica* exposure and milk yield in Holstein cows [20], as well as by reports from Denmark observing a higher prevalence of fasciolosis in milk-oriented Holstein cows compared with other breeds [21, 22]. Given that successful establishment of infection is dependent on both parasite- and host-derived factors, a differential susceptibility of dairy breeds appears most plausible.

As indicated by a previous study from our group on parts of the current data set [20], the objectives of the present study were (I) to investigate the relevance of cattle breed in a parasitological setting in greater detail by estimating associations of *F*. *hepatica*, reflected by farm-level bulk tank milk (BTM) positivity, with production characteristics, i.e. milk yield, milk fat, and milk protein in two different breeds. The underlying hypothesis here was that dairy breeds such as Holstein cows are less resilient to liver fluke infection compared with dual-purpose breeds and hence negative effects on production are more pronounced on Holstein farms. Furthermore, we aimed (II) at evaluating the relevance of production level and potential confounders when assessing the associations of *F*. *hepatica* exposure with production traits. In this context, we hypothesised that irrespective of breed, higher producing farms are more vulnerable to the presence of *F*. *hepatica*, reflected by more profound production losses compared with farms with a relatively lower or medium level of production.

## Materials and methods

### Study farms

Sampling strategy, farm selection, and study population have previously been described in detail [23–25]. Briefly, an extensive, cross-sectional study on animal health, husbandry practices, and farm management was performed in Germany from January 2017 through August 2019. Farms were included in the main dairy areas of the country, i.e. in the north (federal states of Lower Saxony and Schleswig-Holstein), east (federal states of Thuringia, Saxony-Anhalt, Brandenburg, and Mecklenburg-Western Pomerania), and south (federal state of Bavaria). Farms were sampled on the condition to visit 250 farms each in the north, east, and south. Stratification was incorporated within study region by administrative district, herd size, i.e. number of cows, and federal state. Information relevant for sampling was retrieved from the national animal information database (Herkunftssicherungs- und Informationssystem für Tiere, HIT) as well as farm data from the Association for Milk Testing (Milchprüfring Bayern e. V.) and farms were selected implementing an automated approach. Farms were contacted via mail and invited to participate. Subsequently, interested farm managers had to proactively get in touch with the study teams. Farm visits were carried out once throughout the study period. Farm managers granted access to their farm and associated facilities in the context of their voluntary participation in the study. Furthermore, they had the option to make available farm-specific data (e.g. from HIT, and production data) via voluntary written consent. Further permits were not required, since the responsible farm managers decided which permits to grant in the context of voluntary participation in the study. At any given point throughout the study, farmers had the option to draw back from the study or to have specific data excluded from further investigations within the frame of the study. Any personal and farm-specific information was handled in alignment with German and European data protection legislation and according to the consent given by the farm manager.

### On-farm data collection

Farm data were recorded using paper-based questionnaires and data entry forms which were subsequently manually entered into a study database. Farm characteristics, i.e. farming type (conventional/organic) or pasture access, were further recorded during a personal interview with the farm manager as described by Jensen et al. [26]. Production data, i.e. milk yield (in kg), milk fat (in kg), and milk protein (in kg) were retrieved on farm level and adjusted for the number of lactating dairy cows from the national milk recording system (Dairy Herd Improvement, DHI) for the three years period prior to the farm visit. Information on breed was available on individual cow level and retrieved from the national animal information data base (HIT).

### Detection of antibodies against *F*. *hepatica* in bulk tank milk

Towards the end of the grazing season, i.e. August–November, farmers were asked to provide a BTM sample from their farm. This period was chosen in order to increase the comparability among farms. Upon arrival at the laboratory, samples were processed and analysed as described in a previous study [20]. In brief, milk samples were centrifuged (2000 × g, 15 min), skimmed and subsequently stored at -20°C until further processing. The IDEXX Fasciolosis Verification test kit (IDEXX GmbH) was used according to the manufacturer’s instructions to detect antibodies against the f2 antigen of *F*. *hepatica* (sensitivity 95.0%, specificity 98.2% [27]). As recommended by the manufacturer, the threshold for positivity was set at a sample/positive control ratio > 30%. A binary variable (*F*. *hepatica* antibody positive/negative) was created based on the BTM ELISA results.

### Data editing

Plausibility of the data was ascertained on several levels. First, automated plausibility checks based on a-priori determined thresholds were incorporated within the central data base. Secondly, every considered variable was checked for implausible values. If potentially implausible values were detected, the respective part of the data base as well as of the paper-based assessment forms were scrutinised in order to identify whether the implausibility entered during data transcription or export. In case of implausible or missing values, the respective observation was excluded from further analyses. Since data on milk yield, milk fat, and milk protein were available for three years prior to the farm visit, a median value was calculated from the three available values using a non-parametric bootstrap approach with 1,000 resamples with replacement. This allowed for a high conservation of information from the raw values and yielded estimates that reliably reflected the individual farm. This bootstrap condensed the available information taking into account the underlying data based on a likelihood statement rather than a frequency statement and making no assumption about the distribution of the given data.

Furthermore, since values were adjusted for the number of cows per farm, a value on farm level hence reflects the individual cow level, e.g. a certain amount of milk in kg per cow and year. Farms were categorised as German Holstein (GH) or German Simmental (SIM) operations if at least 85% of the cows present on the day of the farm visit were of one of the respective breeds. In cases, where less than 85% of a herd was of a single specific breed, the farm was removed from the data set.

### Statistical analyses

The R Software for Statistical Computing version 4.2.0 and the R Studio interface were used for all analyses [28, 29]. A compilation of implemented packages is provided in S1 Table. Throughout the analyses, statistical significance was set at p ≤ 0.05 and all confidence intervals (CI) were calculated at 95%. Quantile regression [30] was selected as the tool for modelling the associations of *F*. *hepatica* presence with production parameters considering breed. Unlike linear regression, quantile regression estimates the conditional median or quantile of the response and in comparison with ordinary least square regression, quantile regression is more robust to outliers of the target. Moreover, this technique is less strict about model assumptions, e.g. normality of residuals and homoscedasticity [31]. Therefore, quantile regression is more flexible than other regression methods to distinguish differential associations at varying levels of the distribution of the target [32, 33].

Considering the second aim of the present study, i. e. to investigate the relevance of production level or potential confounders, the 25%, 50%, and 75% quantiles of each target variable (i.e. *milk yield*, *milk fat*, *milk protein*) were modelled separately within breed. Hence, the association of the presence of *F*. *hepatica* antibodies was assessed for operations with low (25% quantile), medium (50% quantile), and high (75% quantile) production level within the respective breed.

Quantile regression introduces the idea of quantiles to the context of general linear models [31]. In the present study, the median regression model studies breed-dependent associations on production level:

$$y=\beta_{0}+\beta_{breed}x_{breed}\times \beta_{Fasciola}x_{Fasciola}+\in$$ (1)

where the response *y* is continuous and the predictors *x_breed_* and *x_Fasciola_* are in interaction. As for the models to determine production-level dependent association of *F*. *hepatica* seropositivity with farm level milk yield, milk fat, or milk protein within breed, respectively, the models can be viewed as:

$$y=\beta_{0}+\beta_{Fasciola}x_{Fasciola}+\beta_{i}x_{i}+\ldots \beta_{n}x_{n}+\in$$ (2)

with the 25%, 50%, and 75% quantile of the continuous response *y* being modelled within breed as a function of the predictor *x_Fasciola_* together with potentially present confounding effects of variables *x_i_* and *x_j_*. The R package quantreg [34] was used to construct quantile regression models.

A network structure was drawn using the free software DAGitty [35] in order to identify potential farm-level confounders, i.e. variables with a presumed influence on both the predictors as well as on the response, and to guide the model building process. For the different response variables, network structures are provided in S1–S3 Figs. Subsequently, the identified confounders (*farming type*, *pasture access*, *herd size*) were added to the models. In this very context, one confounding variable was introduced at a time and models were compared using the compare_performance() function from the R package performance [36] as well as by conducting a likelihood ratio test to identify the superior model based on Akaike’s information criterion (AIC) and Bayesian information criterion [37, 38]. If the introduction of a confounding variable improved the quality of the model, i.e. lower values of AIC and BIC, the variable was kept and the estimates of the remaining variables hence were adjusted. To assess the breed-dependent association of *F*. *hepatica* with the response, the emmeans() function from the emmeans package [39] was applied. The issue of multiple comparisons was addressed via the Benjamini-Hochberg procedure to adjust p values [40].

## Results

### Descriptive results

Parts of the descriptive results have been presented elsewhere [20, 24, 25].Data on BTM *F*. *hepatica* antibodies were available for 645 farms. Since 49 farms did not participate in the national milk recording system and 36 farms could not be assigned to one breed, the final data set for analysis consisted of 560 farms housing 93,672 dairy cows in total. The mean herd size was 167 cows with a range from 5–2,821. The majority of farms (n_farms_ = 383; 68.3%) was assigned to the German Holstein breed, and 177 farms to German Simmental (31.6%). Cows were mainly (79.8%) housed in free stall facilities (n_farms_ = 447), followed by tie stall barns (n_farms_ = 54; 9.6%), and other housing types such as pasture-based systems (n_farms_ = 60; 10.7%,). Pasture access was present on 295 operations (52.7%) and 42 farms (7.5%) were managed according to organic farming principles. The presence of *F*. *hepatica* antibodies was confirmed on 81 farms (11.3%). A descriptive overview of the continuous variables is provided in Table 1.

**Table 1:** Descriptive overview of continuous variables in the data set (n_farms_ = 560).

Variable	Mean ± S.D.	Median	IQR	Min–Max
Milk yield^1^^,^ ^2^	8,684.0 ± 1,444.0	8,763.0	2,007.0	3,940.0–12,527.0
Milk fat^1^^,^ ^2^	352.4 ± 54.0	356.4	70.2	161.0–490.3
Milk protein^1^^,^ ^2^	296.9 ± 48.5	301.7	64.5	128.4–412.3
Herd size^3^	167.3 ± 252.0	81.0	142.0	5.0–2,821.0

^1^ Median value per farm.

^2^ in kg.

^3^ number of lactating and dry cows.

### Association of the presence of *F*. *hepatica* with production parameters

**Fig 1:**
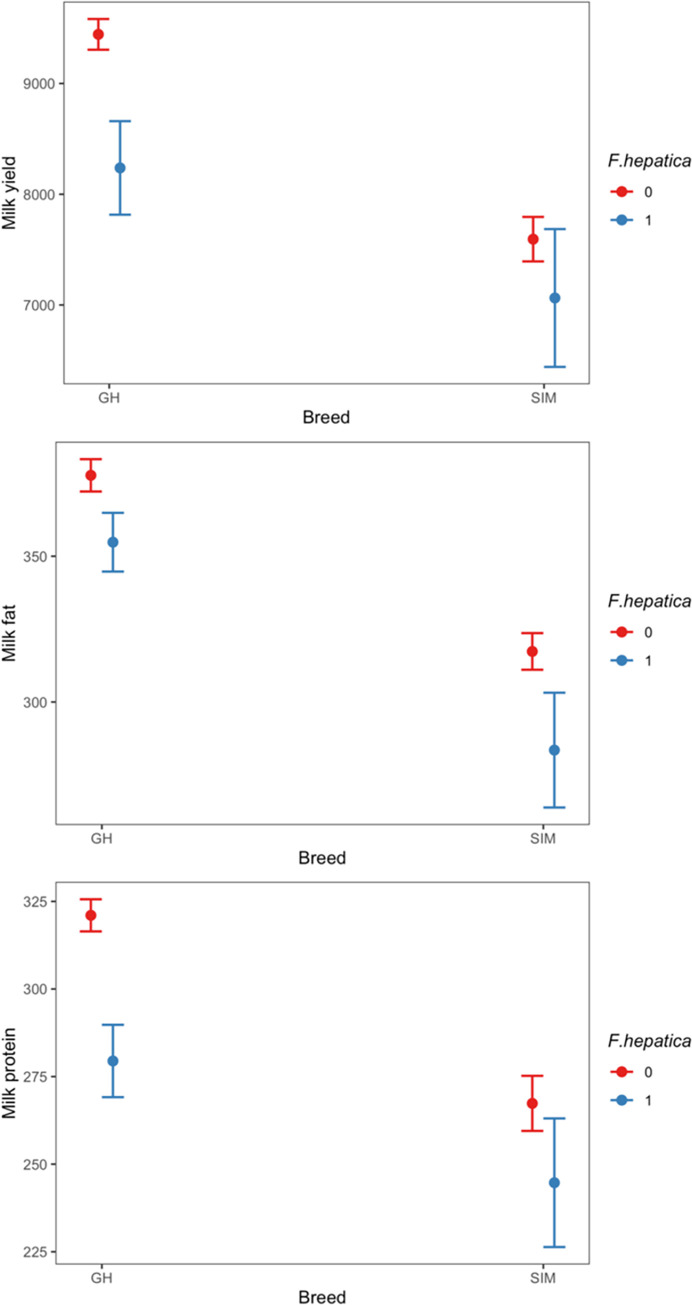
Associations of *Fasciola hepatica* (negative: red; positive: blue) in interaction with breed with milk yield (top, in kg), milk fat (middle, in kg), and milk protein (bottom, in kg), respectively, on 560 dairy farms in Germany. Median values and 95% Confidence Intervals are displayed. GH = German Holstein; SIM = German Simmental.

The results of the first model incorporating an interaction term of breed and the on-farm presence of *F*. *hepatica* antibodies in regard to production parameters are visualised in Fig 1.

On GH farms, the presence of *F*. *hepatica* antibodies entailed a total median reduction of milk yield of 1,206 kg (p < 0.001) with a median milk production of 9,444 kg [95% CI 9,305–9583] on seronegative, and of 8,238 kg [95% CI 7,816–8,660] on seropositive farms. SIM farms had a lower median milk production per se (7,523 [95% CI 7,323–7,723]), but *F*. *hepatica* seropositivity did not appear to be associated with a reduction in milk yield as opposed to GH farms (p = 0.11).

GH farms positive for *F*. *hepatica* antibodies had a lower median milk fat (355 kg [95% CI 345–365]) than *F*. *hepatica* negative farms (378 kg [95% CI 372–383]) which equals a reduction of 22.9 kg milk fat (p = 0.001). On SIM operations, a similar situation was observed with a median reduction of 33.8 kg milk fat (p = 0.01) on seropositive farms (285 kg [95% CI 264–303]) compared with seronegative farms (317 kg [95% CI 311–324]).

A median reduction of milk protein (41.6 kg, p < 0.001) was present on *F*. *hepatica* seropositive GH farms (279 kg [95% CI 269–290]) compared with seronegative farms (321 kg [95% CI 316–326]). The median difference was less pronounced on SIM farms (22.6 kg, p = 0.03) where the parasite was present (245 kg [95%CI 226–263]) compared with farms where *F*. *hepatica* was absent (267 kg [95%CI 259–275]).

### *Fasciola hepatica* and production level within breed

Model results are summarised in Table 2.

**Table 2:** Model results of the associations of Fasciola hepatica seropositivity with production traits within breed across production levels.

Milk yield^1^										
German Holstein										
		Low-yielding farms			Medium-yielding farms			High-yielding farms		
Predictor	Category	Estimate	95% CI	*P*	Estimate	95% CI	*P*	Estimate	95% CI	*P*
Intercept	Continuous	8,932.4	8,643.7–9,221.0	<0.001	9,607.3	9,393.7–9,820.9	<0.001	10,306.5	10,128.4–10,484.6	<0.001
*F*. *hepatica*	Absent	Reference	_	_	_	_	_	_	_	_
	Present	-956.6	-1,330.4 –-582.8	<0.001	-1,115.5	-1,714.9 –-516.1	<0.001	-892.7	-1,460.6 –-324.7	0.02
Farming type	Conventional	Reference	_	_	_	_	_	_	_	_
	Organic	-2,318.3	-2,670.0 –-1,966.7	<0.001	-2,114.3	-3,274.4 –-954.2	<0.001	-1,500.4	-2,490.9 –-509.9	0.003
Pasture access	Absent	Reference	_	_	_	_	_	_	_	_
	Present	-252.8	-611.5–106.0	0.2	-215.5	-500.1–69.1	0.1	-241.4	-494.2–11.3	0.06
German Simmental										
Predictor	Category	Estimate	95% CI	*P*	Estimate	95% CI	*P*	Estimate	95% CI	*P*
Intercept	Continuous	7,153.8	6,957.9–7,349.7	<0.001	7,670.5	7,466.7–7,874.3	<0.001	8,271.0	8,091.9–8,450.2	<0.001
*F*. *hepatica*	Absent	Reference	_	_	_	_	_	_	_	_
	Present	-329.21	-938.4–280.0	0.3	321.3	-467.7–1,110.3	0.4	-154.7	-909.8–600.3	0.7
Farming type	Conventional	Reference	_	_	_	_	_	_	_	_
	Organic	-1,439.6	-1,832.9 –-1,046.2	<0.001	-1,144.4	- 2,042.3 –-245.6	0.01	-1,227.6	-1,413.3 –-1,041.9	<0.001
Pasture access	Absent	Reference	_	_	_	_	_	_	_	_
	Present	-123.4	-750.9–504.2	0.7	-318.4	-942.6–305.8	0.3	174.6	-449.3–798.5	0.6
Milk fat^1^										
German Holstein										
Intercept	Continuous	356.2	347.9–364.4	<0.001	382.3	373.0–391.50	<0.001	412.9	404.8–4.21	<0.001
*F*. *hepatica*	Absent	Reference	_	_	_	_	_	_	_	_
	Present	-27.6	-42.1 –-13.0	<0.001	-21.6	-46.3–3.2	0.09	-30.7	-50.1 –-11.4	0.002
Farming type	Conventional	Reference	_	_	_	_	_	_	_	_
	Organic	-90.5	-101.4 –-79.7	<0.001	-86.3	-127.8 –-44.7	<0.001	-57.6	-87.1 –-28.1	<0.001
Pasture access	Absent	Reference	_	_	_	_	_	_	_	_
	Present	-2.9	-14.4–8.6	0.6	-4.3	-15.5–7.0	0.5	-12.1	-22.7 –-1.6	0.03
German Simmental										
Intercept	Continuous	300.0	290.3–308.8	<0.001	321.3	314.0–328.7	<0.001	343.8	336.0–351.6	<0.001
*F*. *hepatica*	Absent	Reference	_	_	_	_	_	_	_	_
	Present	-15.5	-43.4–12.45	0.3	-0.5	-29.3–28.31	0.97	-13.6	-48.4–21.1	0.4
Farming type	Conventional	Reference	_	_	_	_	_	_	_	_
	Organic	-61.4	-86.7 –-36.2	<0.001	-55.0	-83.0 –-27.1	<0.001	-41.6	-64.6 –-18.5	0.001
Pasture access	Absent	Reference	_	_	_	_	_	_	_	_
	Present	-14.7	-43.2–13.8	0.3	-15.2	-35.3–5.0	0.1	-5.12	-36.6–26.4	0.8
Milk protein^1^										
German Holstein										
Intercept	Continuous	300.0	287.7–311.8	<0.001	323.9	317.0–330.8	<0.001	342.7	332.5–352.9	<0.001
*F*. *hepatica*	Absent	Reference	_	_	_	_	_	_	_	_
	Present	-22.8	-38.3 –-7.3	0.004	-32.1	-50.9 –-13.2	0.001	-20.1	-45.3–5.1	0.1
Farming type	Conventional	Reference	_	_	_	_	_	_	_	_
	Organic	-82.1	-92.0 –-72.3	<0.001	-73.1	-108.3 –-38.0	<0.001	-62.0	-95.1 –-29.0	<0.001
Pasture access	Absent	Reference	_	_	_	_	_	_	_	_
	Present	-8.8	-21.4–3.7	0.2	-9.1	-18.1 –-0.1	0.05	-6.2	-16.9–4.5	0.3
Herd size^2^	Continuous	0.02	-0.01–0.04	0.2	0.01	0.01–0.02	<0.001	0.01	-0.01–0.04	0.3
German Simmental										
Intercept	Continuous	228.8	216.6–241.0	<0.001	252.8	237.5–268.1	<0.001	282.5	267.9–297.0	<0.001
*F*. *hepatica*	Absent	Reference	_	_	_	_	_	_	_	_
	Present	-0.9	-21.3–19.5	0.9	15.3	-13.8–44.4	0.3	-11.3	-34.9–12.4	0.4
Farming type	Conventional	Reference	_	_	_	_	_	_	_	_
	Organic	-55.3	-72.5 –-38.1	<0.001	-64.3	-95.2 –-33.5	<0.001	-40.4	-47.8 –-33.0	<0.001
Pasture access	Absent	Reference	_	_	_	_	_	_	_	_
	Present	-13.9	-37.2–9.3	0.2	-7.8	-33.0–17.4	0.5	2.8	-21.1–26.7	0.8
Herd size^2^	Continuous	0.4	0.3–0.6	<0.001	0.4	0.1–0.6	0.002	0.2	-0.02–0.41	0.07

^1^ in kg

^2^ number of cows

In Holstein farms, the presence of *F*. *hepatica* antibodies entailed a reduction in milk yield irrespective of production level with medium-yielding farms encountering the highest median loss of milk (-1,115.5 kg [95% CI -1,714.9 –-516.1], p < 0.001), followed by low-yielding farms (-956.6 kg [95% CI -1,330.4 –-582.8], p < 0.001), and high-yielding farms (-892.7 kg [95% CI -1,460.6 –-324.7], p = 0.02). Moreover, farming type appeared to be a relevant factor in this model with organic farms having a lower median milk yield level compared with conventional operations (low-yielding farms: -2,318.3 kg [95% CI -2,670.0 –-1,966.7], p < 0.001; medium-yielding farms: -2,114.3 kg [95% CI -3,274.4 –-954.2], p < 0.001; high-yielding farms: -1,500.4 kg [95% CI -2,490.9 –-509.9], p = 0.003). In German Simmental, the presence of *F*. *hepatica* antibodies was not associated with milk production when compared with seronegative farms across production levels. However, farming type was associated with lower milk yield in all three production level categories with organic farming entailing a reduction of 1,439.6 kg ([CI -1,832.9 –-1,046.2], p < 0.001), 1,144.4 kg ([CI -2,042.3 –-245.6], p = 0.01), and 1,227.6 kg ([-1,414 –-1,041.9], p < 0.001) on low-yielding, medium-yielding, and high-yielding farms, respectively, when compared with conventionally run operations.

The milk fat model for the GH breed identified a decreased median milk fat on low-yielding (-27.6 kg [95% CI -42.1 –-13.0], p < 0.001) and high-yielding farms (-30.7 kg [95% CI -50.1 –-11.4], p = 0.002), when *F*. *hepatica* antibodies were present. Furthermore, farming type was a relevant covariate for low-yielding (-90.5 kg [95% CI -101.4 –-79.7], p < 0.001), medium-yielding (-86.3 kg [95% CI -127.8 –-44.7], p < 0.001), and high-yielding (-57.63 kg [95% CI -87.1 –-28.1], p < 0.001) operations, with organic farms consistently displaying a lower median milk fat than conventional farms.

Compared with negative farms, parasite seropositivity was not associated with milk fat in German Simmental across all production levels, but again associations were found with regard to farming type. In contrast to conventional farms, organic farming was associated with lower milk fat on low-yielding (-61.4 kg [CI -86.7 –-36.2], p < 0.001), medium-yielding (-55.0 kg [CI -83.0 –-27.1], p < 0.001), and high-yielding (-41.6 [CI -64.6 –-18.5], p = 0.001) operations.

*Fasciola hepatica* seropositivity appeared to be associated with lower median milk protein on low-yielding (-22.8 kg [95% CI -38.3 –-7.3], p = 0.004) and medium-yielding (-32.1 kg [-50.9 –-13.2], p = 0.001) GH farms. Farming type was relevant throughout production level with organic farming being associated with a median reduction by 82.1 kg ([95% CI -92.0 –-72.3], p < 0.001), 73.1 kg ([95%CI -108.3 –-38.0], p < 0.001), and 62.0 kg ([95% CI -95.1 –-29.0], p < 0.001) on low-yielding, medium-yielding, and high-yielding farms, respectively. Furthermore, an increasing herd size was associated with a median increase of 0.01 kg ([95% CI 0.01–0.02], p < 0.001) milk protein on medium-yielding farms.

The presence of *F*. *hepatica* antibodies was not associated with milk protein on SIM farms. Compared with conventional farming practices, organic farming was associated with lower milk protein on low-yielding (-55.3 kg [CI -72.5 –-38.1], p < 0.001), medium-yielding (-64.3 kg [CI -95.2 –-33.5], p < 0.001) as well as on high-yielding (-40.4 [CI -47.8 –-33.0], p < 0.001) farms. Moreover, larger herd size was associated with higher milk protein on low-yielding (0.4 kg [CI 0.3–0.6], p < 0.001) and medium-yielding (0.4 kg [CI 0.1–0.6], p = 0.002) operations.

## Discussion

Over the past decades, the principal focus of breeding programmes in the dairy sector has been the genetic selection for high milk production levels and an increased output of milk components such as milk fat and milk protein [41–43]. These developments have yielded a type of dairy cow that is capable of producing large amounts of milk. On the downside of these productive advancements, modern dairy cows are characterised by impaired fitness traits [44–46] and lower resistance to disease [47, 48]. Dual purpose breeds, selected for more than one single target criterion, yet have maintained better fitness trait and lower production potential [46, 49]. Holstein cows are an exceptionally specialised breed selected for maximum output. This type of dairy cow is able to fiercely exploit body reserves in order to maintain milk yield and productivity [50–52]. Simmental cows on the other hand represent a dual-purpose breed both for milk and meat production. They have a higher body condition score and greater body muscle mass than breeds that have been exposed to selective pressure for increased milk production. Moreover, Simmental cows appear to mobilise less body reserves, experience lower oxidative stress, and seem to be more capable of correcting the state of negative energy balance [52–54].

As hypothesised and indicated by previous work on parts of this data set [20] the present study revealed differences in the association of *F*. *hepatica* seropositivity with production traits considering breed on the farms studied. When investigating the interaction of breed with the on-farm occurrence of *F*. *hepatica*, the model revealed a considerable median decrease in milk production on *F*. *hepatica* positive GH farms, whereas no such association was evident on SIM farms. Furthermore, a remarkably larger median reduction in total milk protein was observed in *F*. *hepatica* positive GH operations compared with the respective SIM farms where such a decrease appeared less pronounced. Moreover, production level appeared to be of importance in this context which translated into relevant associations in GH herds which were less evident in SIM herds throughout all production levels. Solely in regard to milk fat, *F*. *hepatica* seropositivity was associated with a larger decrease in milk fat in SIM farms than in GH farms. This may likely be explained by the different metabolic reaction towards disturbances present in Simmental and Holstein cows [52–54]. The association of *F*. *hepatica* with production traits has been well-known. Köstenberger et al. [55] and Takeuchi-Storm et al. [56] reported a decrease in milk yield of 6% in *F*. *hepatica* positive herds. A reduction of 15% was observed by Howell et al. [57] in a setting of high prevalence and high-yielding dairy herds. Yet, a decrease of 18–32% has been described in low-producing herds as well [58]. Takeuchi-Storm et al. [56] have explained that a reduction of 580.8 kg of milk as reported in their study is estimated to be even higher than the 375 kg reduction due to clinical mastitis and associated effects [59]. Considering the fact that infections with the common liver fluke may persist for more than two years, the chronicity of hepatic changes and the economic implications may well be substantial [60]. Equivocal evidence has been presented regarding the association of *F*. *hepatica* with milk fat and milk protein. In contrast to the current results, several studies were not able to acknowledge relevant associations with milk fat [57] or milk protein [8, 57]. It yet remains plausible to detect an association of this kind, given that *F*. *hepatica* may well interfere with energy metabolism via reduced feed intake, impaired feed-conversion and an overall compromised liver function as a consequence of pathological changes induced by migrating and resident flukes [61–63]. Interestingly, when considering production level of the analysed farms and the role of potential confounders in this setting of covariates, it became evident that *F*. *hepatica* was not associated with a reduction of all three production characteristics considered in SIM farms, whereas effects where still observable in GH farms. When comparing the 95% CI for the associations to see if confidence intervals overlap, indicating a breed dependence, the results point out that breed may be a discriminating aspect in this context. Regardless, potential breed-dependent effects are to some degree mediated by production level. Our results hence lend support to the idea of the relevance of breed regarding the association of *F*. *hepatica* with production parameters and may indicate potentially higher tolerance, i.e. an increased ability to maintain production even in the presence of infection, in SIM cattle [64]. This coincides with observations by Hayward et al. [65], who reported variation between breeds in regard to the severity of liver fluke infection, and is well in alignment with extant literature on inherent differences among breeds in their susceptibility to disease [66, 67]. The present study hence may well serve as a starting point for digging deeper into his matter in order to understand possibly varying levels of host-parasite interactions in different dairy breeds.

Organic farming was consistently associated with reduced milk yield, lower milk fat as well as lower milk protein across breeds and production levels. This is in alignment with previous research indicating that milk production is up to 35% lower in organic than in conventional herds [68–70], which is probably attributable to lower energy intake via less concentrate or forage in an organic farm setting [69–71]. As for milk fat, equivalent evidence has been provided on the differences between organic and conventional operations [72]. Several authors have traced back increased milk fat in organic milk to the fact that dairy breeds other than Holstein are used on organic farms which may subsequently translate into a higher milk fat [73–75]. Moreover, concentrate-rich diets entailing a decline in milk fat are common on conventionally managed dairy farms [72, 76]. Other authors hypothesised that lower milk fat in organic milk compared with conventional milk could be a result of fat supplement enriched diets on conventional farms [77] or negative energy balance on organic farms [78]. Similarly, ambivalent results have been discussed for the comparison of milk protein between conventional and organic farms [72]. Whereas Bilik and Łopuszańska-Rusek [68] as well as Sundberg et al. [79] reported higher amounts of milk protein in conventionally produced milk, Vicini et al. [80] found a higher protein concentration in organic milk. Both observations may be the result of the corresponding feeding regime and breed composition of the farms [72]. Pasture access appeared to be associated with reduced milk fat in high-yielding GH farms as well as with lower milk protein in medium yielding GH farms only. Pasture being a relevant factor is probably attributable to the specific setting on these farms and can further be explained with the aforementioned, since pasture access is an integral part of organic farming.

Increasing herd size was associated with a slightly higher milk protein in medium-yielding GH farms as well as on low- and medium-yielding SIM farms. Differences in production with herd size have been described by other authors [81, 82]. Herd size most likely serves as a proxy for the interplay of several factors related to housing and management.

Some bias might have entered the study through voluntary participation of farmers. This may have encouraged proactive managers to be overrepresented in the study population. Assuming that proactive farmers could be more open to external consultation, they may equally be more aware of potential improvement areas on their operations. Hence, the study population may have consisted of farms with an above average health situation. On the contrary, farms specifically seeking assistance with ongoing issues of animal health and husbandry on their farms might have been more inclined to be enrolled to the current project. Consequently, farms with a lower standard of animal health and management procedures than in the underlying population of dairy operations might be overrepresented in the current data set. We cannot exclude selection bias due to the setting the study could be conceived in, but we assume that it is relatively minor due to the rigorous randomisation process and the alignment of outcomes with the extant literature. Cross-sectional studies do not permit the inference of causalities among assessed variables [83, 84]. To assess the true nature of the interaction of *F*. *hepatica* with production parameters and the mechanisms by which the parasite interacts with different host breeds are yet to be explored using specific study designs to draw causal conclusions. Sample size calculation for data collection had been performed stratified by study region. In the current work, analyses yet were conducted in a cross-regional manner. The main reasons for stratified sample size calculation were different farm densities and differing herd size distributions across regions. For example, study region East is characterised by the predominance of fewer and larger, industrialised dairy operations whereas farm density in study region South is considerably higher and farms are mainly family-run facilities [85]. Furthermore in this context, it is important to mention that the distribution of breeds differs considerably across regions with Holstein being the predominant dairy breed in regions North and East and Simmental being the main breed in region South [85]. Hence, different breeds are also to some extent managed differently (e.g. differences in grazing management and potentially also anthelmintic treatment) which may subsequently translate into the parasitological situation. To acknowledge the underlying sampling strategy as well as the fact that differences across regions may be mediated via breed or other potential confounding factors, we incorporated several factors that had initially been considered to reflect region-specific traits or confounders for breed such as e.g. herd size and pasture into our models. Moreover, in analyses prior to this work, the relevance of region had been evaluated by including region as a fixed effect in the models. Since this has not led to major influences on model results, we concluded that in the current analysis, the relevance of region was not substantial for modelling which emphasises the epidemiological soundness of the present work.

When using antibodies to measure *F*. *hepatica* status, the fact that different breeds are sampled opens up the possibility of differential immune responses in Holstein animals compared with other breeds such as Simmental. It is beyond the scope of the present study to further explore this topic, but the results may lend support to the idea of this being worth investigating further. Since we cannot exclude potential confounding factors as a result of varying immune response depending on breed, this needs to be taken into consideration when interpreting the results of this study.

Profitability on a dairy farm is largely determined by productivity [86]. Apart from milk yield, milk fat and milk protein are critical criteria for the quality of the produced milk. Milk fat as the principal energy component of dairy products is a factor relevant for the quality, palatability, and flavour of milk [86–88]. Total milk protein is an important aspect for processing and hence a high protein in raw milk is preferred by dairy plants [86]. Even though the incidence of parasitic diseases might be relatively low, the affiliated economic losses may well be substantial especially since they impact several aspects of dairy cow productivity [7, 89]. Given the present challenges in combating bovine fasciolosis, selecting for breeds more tolerant to the implications of parasite colonisation may be a promising perspective to consider. This is emphasised by the fact that prevalences remain high and the current control measures such as anthelmintic treatment are sub-optimal or not sustainable in the face of upcoming flukicide resistance [90–92]. Since parasitism is always driven by both parasite- and host-associated factors, breeding programmes targeting the improvement of disease tolerance may be an innovative, seminal opportunity.

## Supporting information

S1 Fig Network structure for identification of confounders for the target variable Milk yield. Variables and their presumed relationships among on each other, with the target (farm level milk yield), and with the predictors (farm level status for *Fasciola hepatica*, breed) are represented in nodes. I (blue): target variable; green: variables and predictors; red: confounders; black arrows: relationship among predictors and other variables without involvement of the target; green arrows: relationships involving the target; red arrows: involvement of confounding variables; arrowhead in both directions: presumed association; arrowhead in one direction: presumed influence.
(TIF)

S2 Fig Network structure for identification of confounders for the target variable Milk fat. Variables and their presumed relationships among on each other, with the target (farm level milk fat), and with the predictors (farm level status for *Fasciola hepatica*, breed) are represented in nodes. I (blue): target variable; green: variables and predictors; red: confounders; black arrows: relationship among predictors and other variables without involvement of the target; green arrows: relationships involving the target; red arrows: involvement of confounding variables; arrowhead in both directions: presumed association; arrowhead in one direction: presumed influence.
(TIF)

S3 Fig Network structure for identification of confounders for the target variable Milk protein. Variables and their presumed relationships among on each other, with the target (farm level milk protein), and with the predictors (farm level status for *Fasciola hepatica*, breed) are represented in nodes. I (blue): target variable; green: variables and predictors; red: confounders; black arrows: relationship among predictors and other variables without involvement of the target; green arrows: relationships involving the target; red arrows: involvement of confounding variables; arrowhead in both directions: presumed association; arrowhead in one direction: presumed influence.
(TIF)

S1 Table Compilation of implemented packages. R packages used in the present study.
(DOCX)

S2 Table Analysed data set. (XLSX)
