# Supplementary figures and images for: Associations of production characteristics with the on-farm presence of Fasciola hepatica in dairy cows vary across production levels and indicate differences between breeds
Source: PLoS One. 2023 Nov 17;18(11):e0294601. doi: 10.1371/journal.pone.0294601 (PMC10656002; doi:10.1371/journal.pone.0294601)

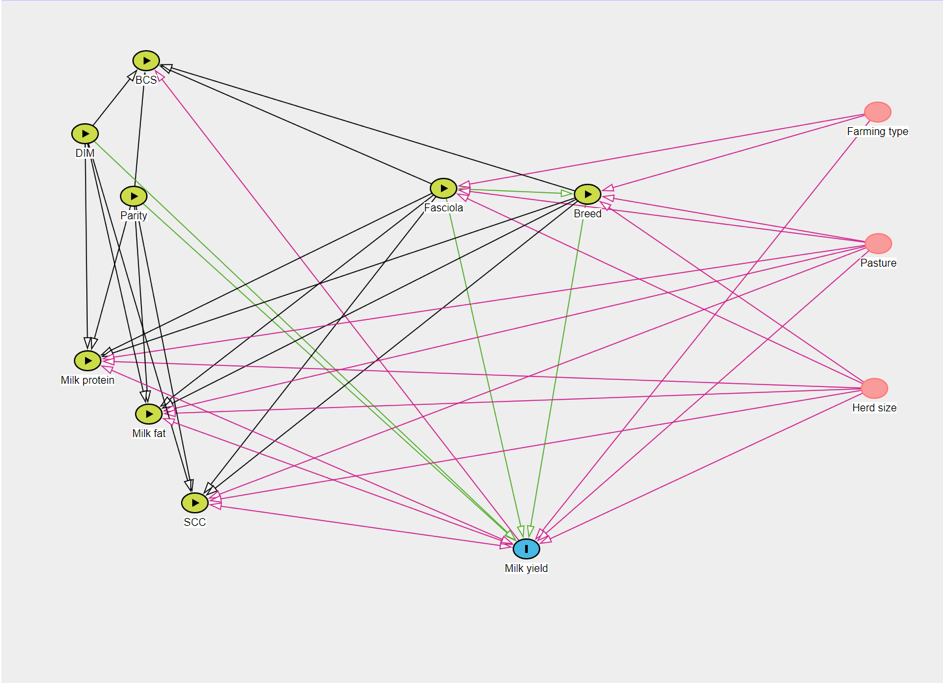

Supplement: S1 Fig — Variables and their presumed relationships among on each other, with the target (farm level milk yield), and with the predictors (farm level status for Fasciola hepatica, breed) are represented in nodes. I (blue): target variable; green: variables and predictors; red: confounders; black arrows: relationship among predictors and other variables without involvement of the target; green arrows: relationships involving the target; red arrows: involvement of confounding variables; arrowhead in both directions: presumed association; arrowhead in one direction: presumed influence. (TIF) [file pone.0294601.s001.tif]

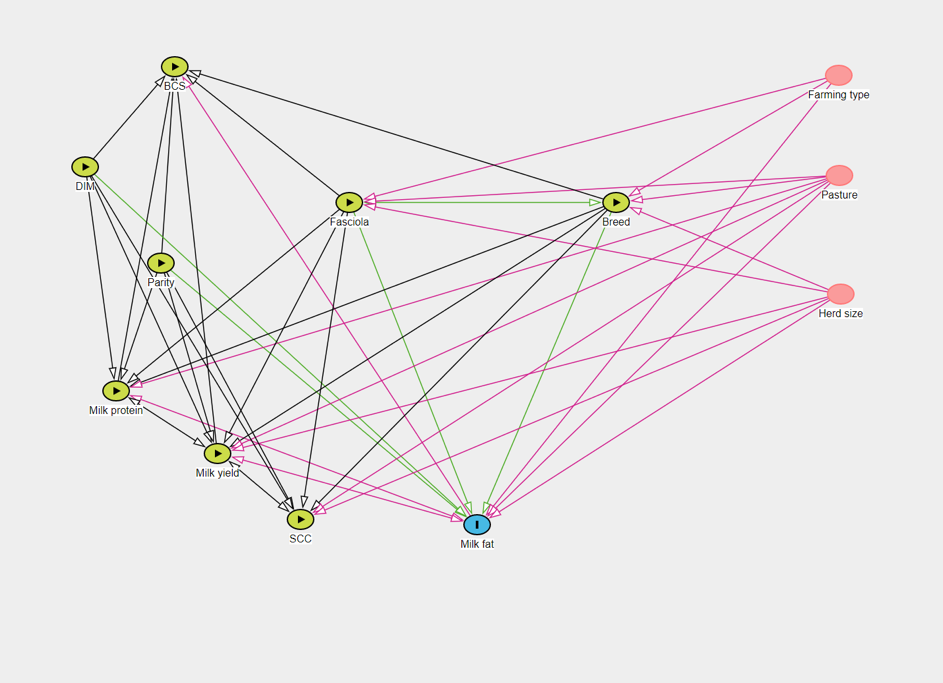

Supplement: S2 Fig — Variables and their presumed relationships among on each other, with the target (farm level milk fat), and with the predictors (farm level status for Fasciola hepatica, breed) are represented in nodes. I (blue): target variable; green: variables and predictors; red: confounders; black arrows: relationship among predictors and other variables without involvement of the target; green arrows: relationships involving the target; red arrows: involvement of confounding variables; arrowhead in both directions: presumed association; arrowhead in one direction: presumed influence. (TIF) [file pone.0294601.s002.tif]

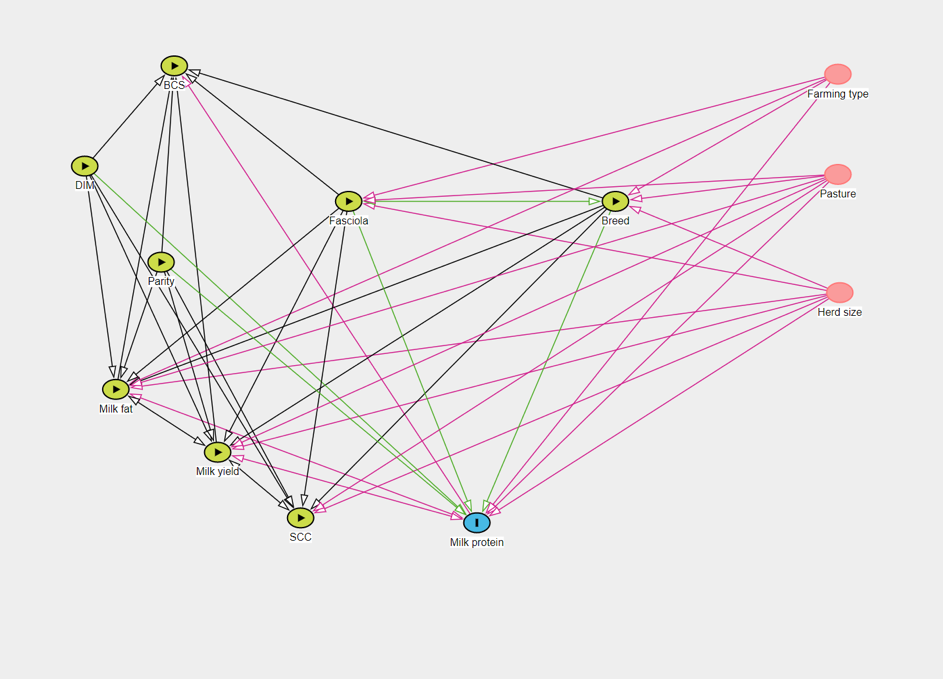

Supplement: S3 Fig — Variables and their presumed relationships among on each other, with the target (farm level milk protein), and with the predictors (farm level status for Fasciola hepatica, breed) are represented in nodes. I (blue): target variable; green: variables and predictors; red: confounders; black arrows: relationship among predictors and other variables without involvement of the target; green arrows: relationships involving the target; red arrows: involvement of confounding variables; arrowhead in both directions: presumed association; arrowhead in one direction: presumed influence. (TIF) [file pone.0294601.s003.tif]
