# Supplementary material for: Associations of production characteristics with the on-farm presence of Fasciola hepatica in dairy cows vary across production levels and indicate differences between breeds
Source: PLoS One. 2023 Nov 17;18(11):e0294601. doi: 10.1371/journal.pone.0294601 (PMC10656002; doi:10.1371/journal.pone.0294601)
Supplement: S1 Table — R packages used in the present study. (DOCX) [file pone.0294601.s004.docx]

**Table 1.** R packages used in the present work.

| **Name of package** | **Reference** |
| --- | --- |
| car | [1] |
| effects | [2] |
| emmeans | [3] |
| lindia | [4] |
| lmTest | [5] |
| lme4 | [6] |
| lmerTest | [7] |
| MASS | [8] |
| magrittr | [9] |
| performance | [10] |
| plyr | [11] |
| quantreg | [12] |
| vcd | [13] |

# **References**

1. Fox J, Weisberg S. An R Companion to applied regression. Thousand Oaks, CA, USA: Sage; 2019.

2. Fox J, Weisberg S. Visualizing fit and lack of fit in complex regression models with predictor effect plots and partial residuals. J Stat Soft. 2018;87:1-27.

3. Lenth R. emmeans: Estimated marginal means, aka least-squares means. R package version 1.7.5. 2022.

4. Lee Y, Ventura S. lindia: Automated linear regression diagnostic. R package version 0.9. 2017.

5. Zeileis A, Hothorn T. Diagnostic checkin in regression relationships. R News. 2002;2:7 10.

6. Bates D, Maechler M, Bolker B, Walker S. Fitting linear mixed-effects models using lme4. J Stat Softw. 2015;67:1-48.

7. Kuznetsova A, Brockhoff PB, Christensen RHB. lmerTest package: Tests in linear mixed effects models. J Stat Softw. 2017;82:1-26.

8. Venables W, Ripley B. Modern applied statistics with S. Fourth ed. New York, NY, USA: Springer; 2002.

9. Bache S, Wickham H. magrittr. a forward-pipe operator for R. R package version 2.0.3. 2022.

10. Lüdecke D, Mattan S. performance: An R package for assessment, comparison, and testing of statistical models. J Stat Softw. 2021;6:3139.

11. Wickham H, Francois R, Henry L, Müller K. dplyr: A grammar of data manipulation. R package version 1.0.7. 2021.

12. Koenker R. quantreg: Quantile regression. R package version 5.93. 2022.

13. Meyer D, Zeileis A, Hornik K. vcd: Visualizing categorical data. R package version 1.4 10. 2022.
